# 

**Published:** 1853-08

**Authors:** 


### CONTENTS

### OF NO II.

### ORIGINAL COMMUNICATIONS.

### ESSAYS AND CASES.

Art. I.—Remarks on the Cause and Treatment of Cholera Infantum. By L. P. Yandell, M. D., Professor of Physiology and Pathological Anatomy in the University of Louisville. - - - - - - - 93

Art. II.—A Memoir of Dr. Charles Caldwell. By. L. P. Yandell, M. D. - - - - - - 101

Art. III.—A case of obstinate and prolonged rigidity, with spasmodic contraction of the neck of the Uterus during parturition, treated by the combined administration of Ergot and Chloroform. By Napoleon B. Anderson, M. D., of Louisville, Ky. - - - - - 114

Art. IV.—Hints with regard to the natural history and medicinal properties of Veratrum Viride. By Isaac Branch, M. D., of Abbeville, S. C. - - - - - 120

### REVIEWS.

Art. V.—A Treatise on Lightning Conductors; compiled from a work on Thunderstorms, by S. W. Harris, F. R. S., and other standard authors. By Lucius Lyon, A. M. 124

### SELECTIONS FROM FOREIGN AND HOME JOURNALS.

Scarlet Fever. - - - - - - - - 141

Wounds of the Heart. - - - - - - - 145

Pills of Sulphate of Quinine. - - - - - - - 154

Recent Researches of Prof. Agassiz. - - - - - 156
Remarkable case of Catalepsy. - - - - - 158
Coffee-Leaves as a Substitute for Tea. - - - - - 161
Encysted Calculus. - - - - - - - 164
Color-Blindness. - - - - - - - 170
Veratrum Viride. - - - - - - - 171
Duty of Medical Men. - - - - - - 174
Stomatitis Materni. - - - - - - - 175

ORIGINAL INTELLIGENCE.

Tribute to Dr. Caldwell. - - - - - - 177
The Fishes of the United States. - - - - - 178
Experimental Investigation Table-moving. - - - 181
Circular Medical Association. - - - - - 184

